# Supplementary material for: Ubiquitin-Specific Protease 2 (OsUBP2) Negatively Regulates Cell Death and Disease Resistance in Rice
Source: Plants (Basel). 2022 Sep 29;11(19):2568. doi: 10.3390/plants11192568 (PMC9571816; doi:10.3390/plants11192568)
Supplement: Supplementary file 1 [file plants-11-02568-s001.zip › plants-1899998-supplementary.pdf]

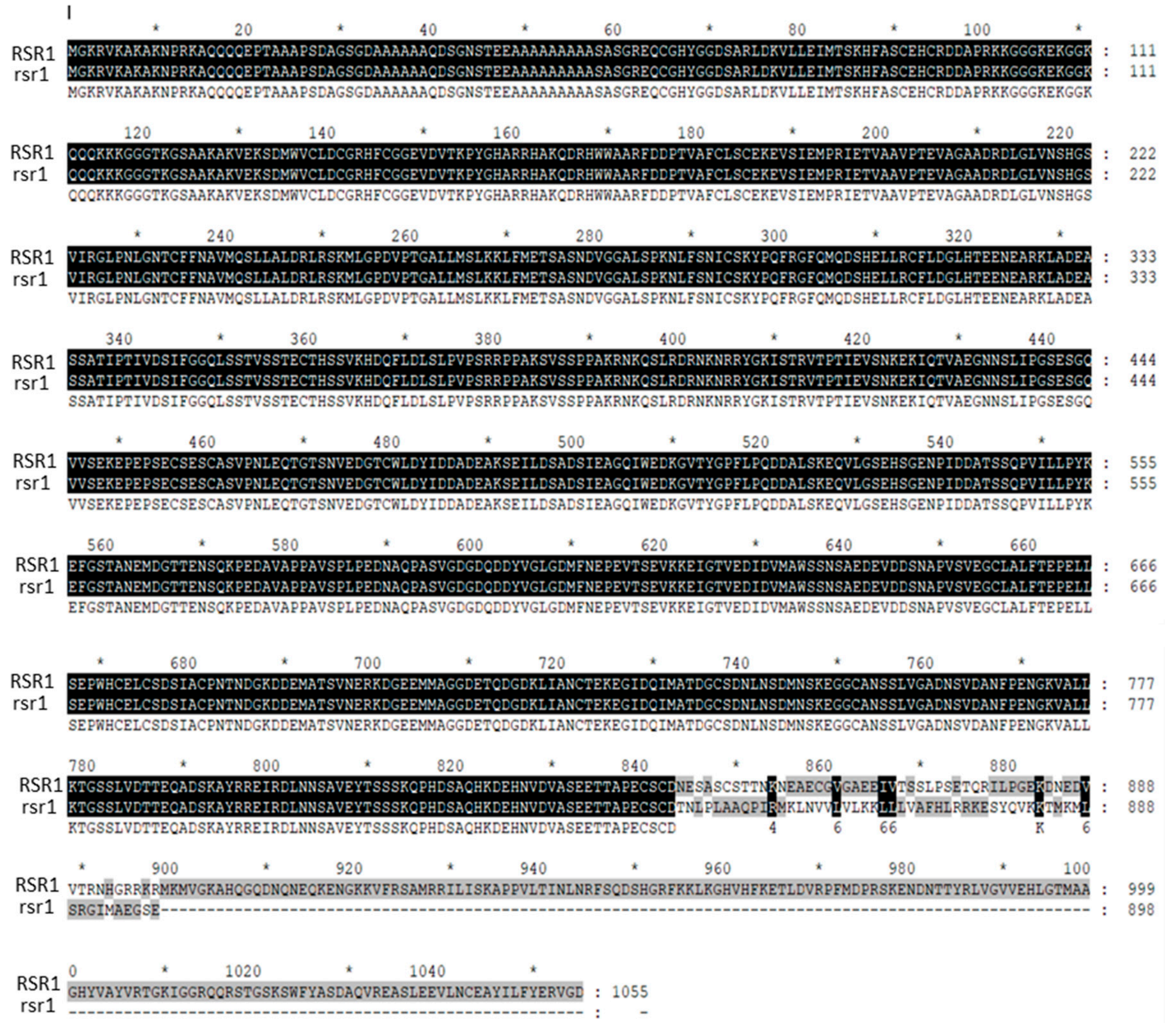

Supplemental Figure S1. Protein sequence alignment of RSR1 and rsr1. rsr1 protein has a shift mutation starting at amino acid 845 and terminating translation at amino acid 899 compared to RSR1 protein.

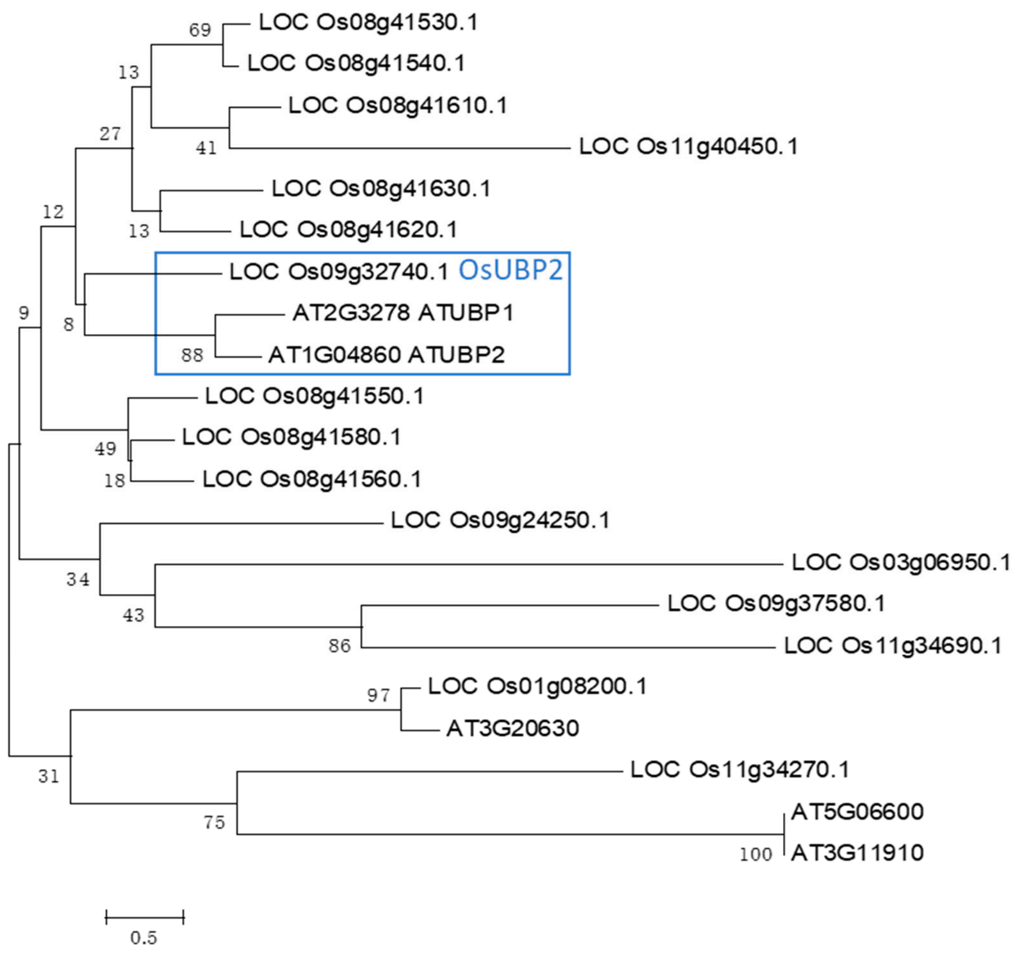

Supplemental Figure S2. Phylogenetic tree of OsUBP2 homologous proteins in rice and Arabidopsis. The tree was generated in MEGA 5 using the minimal evolution method with 1000 boots trap tests. The numbers at the nodes refer to bootstrap support percentage. The length of the branch lines indicates the extent of divergence according to the scale (relative units) at the bottom. At: Arabidopsis thaliana, Os:Oryza sativa. All sequences were downloaded from NCBI (https://www.ncbi.nlm.nih.gov/).

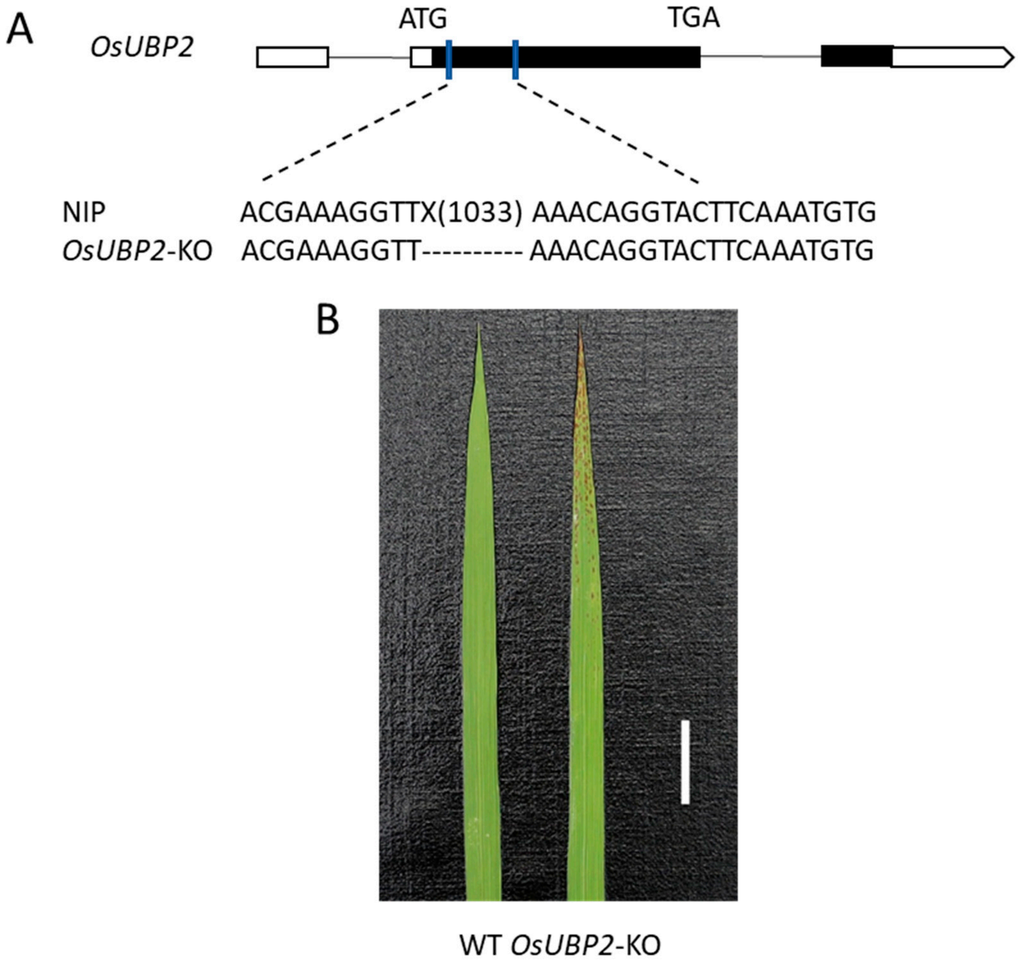

Supplemental Figure S3. The phenotype of *OsUBP2*-KO plant. (A) OsUBP2-KO plant with a 1033bp nucleotide deletion between 370 and 1403 (start from ATG). (B) Leaves of WT and *OsUBP2*-KO plants at the seedling stage. Bar = 2 cm.

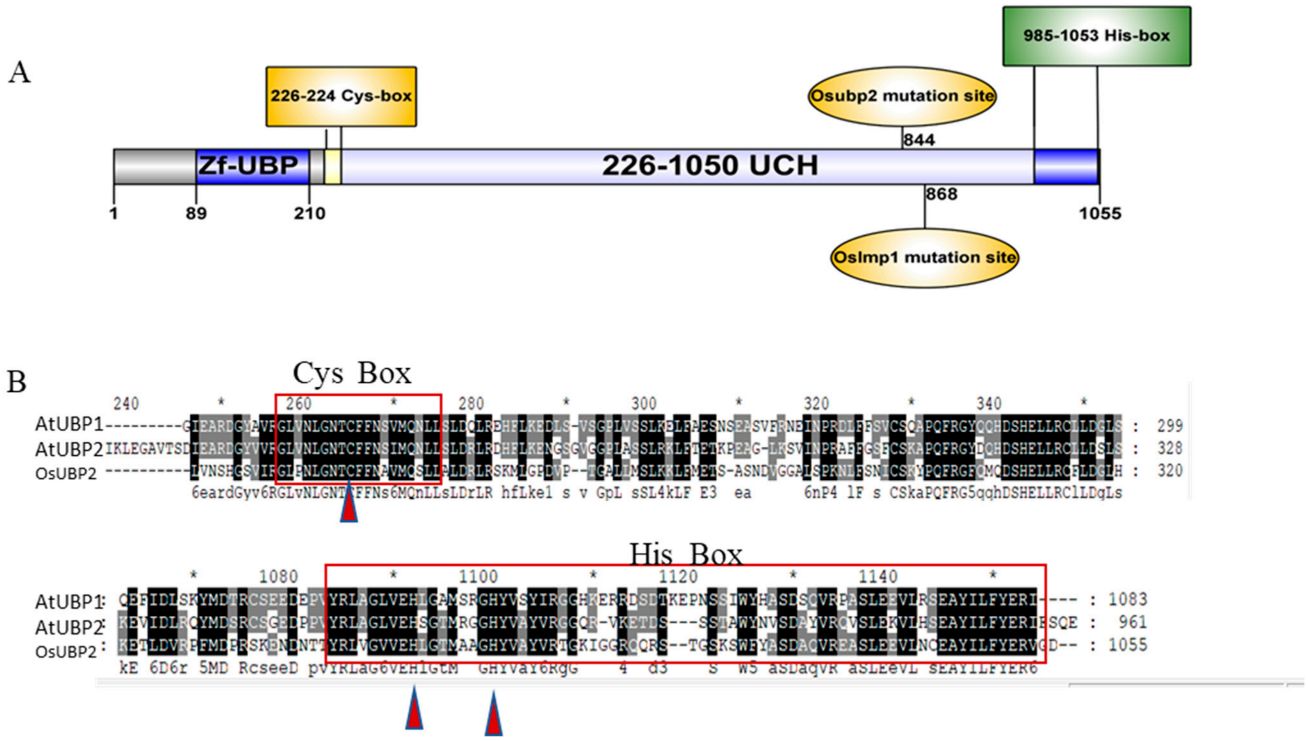

Supplemental Figure S4. The conserved structural domains (A) and catalytic motifs (B) of UBP2 protein. (A) Conserved domains of OsUBP2 protein. The structure was drawn using DOG software. B, Comparison of the deduced amino acid sequences of OsUBP2 and AtUBP1 and AtUBP2. The number in the figure A represent the position of the amino acid. The number in the figure Brepresent the position of the amino acid in AtUBP1. At: *Arabidopsis thaliana*, Os:Oryza sativa. All sequences were downloaded from NCBI (https://www.ncbi.nlm.nih.gov/).

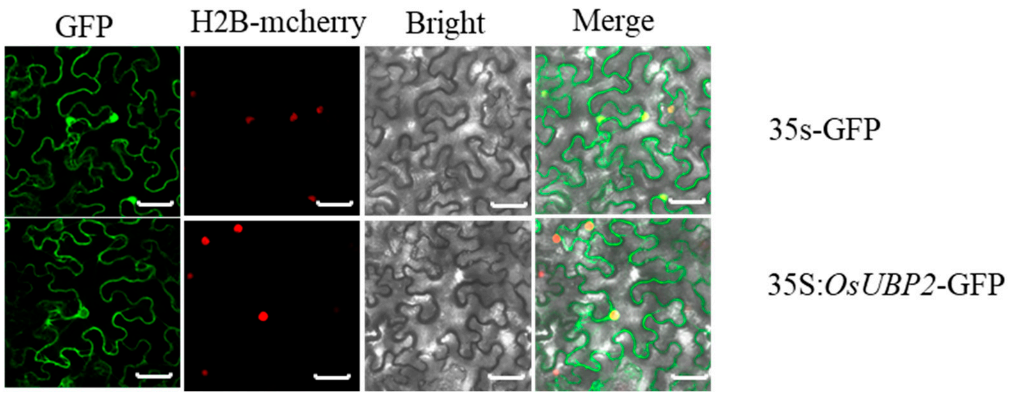

Supplemental Figure S5. Subcellular localization of OsUBP2. *Agrobacteria* containing plasmids of 35S-OsUBP2-GFP/GFP were infected in *N. benthamiana* with H2B-mCherry (localized in nuclear). The GFP/mCherry signal was evaluated via confocal microscopy at 96 h after *Agrobacteria* infection. Bars = 20 μm.

Supplemental Table S1. F_2 generation segregation ratio after crossing the *rsr1* mutant with *Kasalath*

WT Phenotype	Lesion Phenotype	χ2
114	36	0.777297

Supplemental Table S2.

Marker	Sense primer (5’-3’)	Antisense primer (5’-3’)
17.7MB	CAGTTCCGAGCAAGAGTACTC	GGATCGGACGTGGCATATG
19.4MB	TTTGCACTGACTAAGTAGCATC	CGGTCTCTACATCCAACCAT
19.7MB	CATAGTGGAGTATGCAGCTGC	CCTTCTCCCAGTCGTATCTG
RM107	AGATCGAAGCATCGCGCCCGAG	ACTGCGTCCTCTGGGTTCCCGG
RM6174	TCGAGGTGGAGAAGCAGC	TAGTCTTCGTGTCACGCAGC
